# Artificial Intelligence in Clinical Supervision and Training of Psychologists and Other Mental Health Professionals: A Scoping Review

**DOI:** 10.1002/cpp.70359

**Published:** 2026-11-09

**Authors:** Jorge Victor Nascimento da Silva, Paulo Vinicius Silva Ferreira, Pedro Paulo Salustiano dos Santos, Maria Amélia Penido

**Affiliations:** ^1^ Federal Center for Technological Education of Rio de Janeiro (CEFET/RJ) Rio de Janeiro RJ Brazil; ^2^ Pontifical Catholic University of Rio de Janeiro (PUC‐Rio) Rio de Janeiro RJ Brazil

**Keywords:** artificial intelligence, clinical supervision, mental health professionals, psychotherapy training, scoping review, therapist competence

## Abstract

**Background:**

Clinical supervision is fundamental to the development of competent mental health professionals; however, established approaches are limited by challenges in scalability, direct observation and timely feedback. Artificial intelligence has emerged as a potential adjunct to supervision and clinical training, yet the full scope of existing applications has not been systematically mapped.

**Objective:**

This scoping review systematically mapped artificial intelligence applications in the clinical supervision and training of psychologists and other mental health professionals.

**Methods:**

The review was conducted in accordance with Joanna Briggs Institute guidance and reported following the PRISMA‐ScR. The protocol was registered on the Open Science Framework. Searches were conducted in nine databases, with supplementary searches in Google Scholar. Two reviewers independently screened records; charted data were checked against the original reports.

**Results:**

Database searches identified 11,975 records; 4381 duplicates were removed, 7594 records were screened, 817 reports were assessed for eligibility and 73 sources of evidence were included. Four application categories were identified: virtual patient simulation, automated performance assessment, artificial intelligence‐enhanced supervisory feedback and training analytics. Most studies were published from 2022 onward and were concentrated in structured therapies, university‐based settings and high‐income countries.

**Conclusions:**

Artificial intelligence is best positioned as an adjunct that expands opportunities for practice, observation and structured feedback, rather than as a replacement for human supervision. Future research should employ more rigorous designs, validated competency measures, transparent reporting of artificial intelligence systems and outcomes relevant to trainees, supervisors and clients.

**Summary**

- Current AI applications in clinical training are concentrated in simulated clients, automated competency assessment, supervisory feedback and training analytics.
- AI may support practice and structured feedback, but increased self‐efficacy and satisfaction should not be interpreted as demonstrated clinical competence.
- Human supervisors remain responsible for case conceptualization, supervisee anxiety and distress, transference and countertransference, clinical risk and ethical and cultural judgement.
- Training programs implementing AI should establish governance structures for privacy, bias monitoring, data security, trainee support and escalation procedures to human supervisors.
- Evaluate observed therapeutic performance and transfer to clinical practice separately from confidence, diagnostic recognition and the accuracy of AI‐generated scores.

## 1 Introduction

Mental health systems internationally are under sustained strain. The Global Burden of Disease Study estimates that depression and anxiety disorders increased by more than 25% during the early COVID‐19 pandemic (World Health Organization 2022). Demand for qualified mental health professionals consistently surpasses supply, particularly in low‐ and middle‐income countries, where the treatment gap for common mental disorders exceeds 75% (Hanna et al. 2018; Hoeft et al. 2018).

Clinical supervision supports trainees in developing competence, integrating evidence‐based practices into clinical work and managing the demands of practice (Bradley and Becker 2021; Choy‐Brown and Stanhope 2018; Iosim et al. 2021). Its effects on client outcomes remain uncertain: a recent meta‐analysis found a small, statistically non‐significant pooled effect on patient symptoms (Schreyer et al. 2025). Reviewing session recordings and assessing treatment fidelity can require substantial staff time and expertise (Marriott et al. 2026). When supervision relies on supervisees' accounts alone, aspects of clinical practice may remain unobserved and trainees may withhold relevant information (Choy‐Brown and Stanhope 2018; Foskett and Van Vliet 2021). In low‐resource settings, limited access to psychotherapy‐trained supervisors creates additional barriers to structured supervision (Rimal et al. 2025).

These limitations have substantial practical consequences. In routine community mental health care, supervision time may be available while direct observation and other feedback activities recommended in best‐practice models remain less consistently reported (Choy‐Brown and Stanhope 2018). Monitoring adherence to evidence‐based protocols is difficult when reviewing and coding session recordings requires time and trained staff (Marriott et al. 2026). Generative AI has been proposed as a way to supplement psychiatric education, including simulation and assessment, but evidence of educational effectiveness remains limited (Lee et al. 2025).

### 1.1 Artificial Intelligence in Mental Health Education

Artificial intelligence (AI) has been explored for diagnostic support, monitoring and psychoeducation in mental health (Olawade et al. 2024). Applications relevant to training include automated evaluation of psychotherapy material (Hirsch et al. 2018), fidelity feedback (Creed et al. 2022) and structured practice of counselling responses (Tanana et al. 2019). Large language models (LLMs) also enable simulated client conversations (Holderried et al. 2026; Sawah et al. 2026). These applications differ in their proximity to supervision: some evaluate an algorithm or a simulated task, whereas others are embedded in clinical training. Technical feasibility alone does not establish educational effectiveness.

### 1.2 Limitations of Generative AI and LLMs

Generative AI introduces limitations that are directly relevant to clinical training. LLMs generate text from patterns learned during training; fluent output does not establish factual accuracy or clinical understanding (Bender et al. 2021). Training applications therefore require evaluation of both their outputs and the consequences of using those outputs in learning.

Incorrect or clinically inappropriate feedback could reinforce errors in practice. Bias is also a concern: Bouguettaya and Aboujaoude (2025) found racial differences in psychiatric recommendations generated by LLMs under otherwise comparable case conditions. Finally, conversational fluency can encourage users to attribute relational capacities to a system that has no professional responsibility for the interaction. Ethical analyses have raised concerns about artificial empathy, attachment, privacy and accountability in mental health applications (Fiske et al. 2019). These issues warrant human review and explicit investigation in training settings.

### 1.3 Prior Reviews and the Knowledge Gap

Previous reviews provide complementary perspectives. Magill et al. (2022) mapped technology‐based counselling skills training before the widespread availability of LLMs. Aafjes‐van Doorn et al. (2021) examined machine learning across psychotherapy research and Laricheva et al. (2026) examined NLP applications in counselling and psychotherapy. More recent reviews addressed AI in psychiatric education and in psychiatry and psychology education (Lee et al. 2025; Prégent et al. 2025; Weightman et al. 2025). The present review focuses on applications relevant to supervision and professional training, with particular attention to what their reported outcomes can establish about learning and clinical competence.

This review addresses these gaps by systematically mapping AI applications in the supervision and training of psychologists and other mental health professionals, with particular focus on the period following the widespread availability of LLM‐based tools. A preliminary search of PROSPERO, PubMed, the Cochrane Database of Systematic Reviews and JBI Evidence Synthesis confirmed the absence of current or in‐progress reviews on this specific topic. The review therefore asks: What are the existing applications of AI in the clinical supervision and training of psychologists and other mental health professionals? The objectives were to identify the range of AI applications in supervision and training contexts, map relevant training contexts and psychological domains, characterize AI technologies and reported functionalities and identify gaps calling for further investigation.

## 2 Methods

### 2.1 Study Design

Given that this review aimed to identify and map the available literature, a scoping review methodology was considered most appropriate (Munn et al. 2018). The review was conducted in accordance with methodological guidance for scoping reviews established by the Joanna Briggs Institute (JBI) and reported following the Preferred Reporting Items for Systematic Reviews and Meta‐Analyses extension for Scoping Reviews (PRISMA‐ScR).

### 2.2 Protocol and Registration

The protocol for this review was registered on the Open Science Framework platform (https://osf.io/h8473/) to ensure methodological transparency. The results were reported in accordance with the Preferred Reporting Items for Systematic Reviews and Meta‐Analyses extension for Scoping Reviews (PRISMA‐ScR), including a flow diagram detailing the study selection process (Tricco et al. 2018).

### 2.3 Research Question

The central research question, structured using the PCC acronym, was: What are the existing applications of AI in the clinical supervision and training of psychologists and other mental health professionals?

Population (P): psychologists in clinical supervision, supervisors and other mental health professionals in training.

Concept (C): Applications of AI (e.g., machine learning, natural language processing, chatbots, and generative language models) in clinical training and supervision.

Context (C): Implementation in scholarly or occupational clinical training settings.

### 2.4 Eligibility Criteria

#### 2.4.1 Participants

Studies were eligible if they included psychologists in clinical supervision, supervisors responsible for professional development, or other mental health professionals in training. This included psychologists at initial or intermediate stages of clinical practice, residents or interns in specialization or multidisciplinary residency programs, counsellors in training and supervisors who monitored and evaluated trainee progress.

#### 2.4.2 Concept

The review encompassed the set of AI applications, including machine learning techniques, natural language processing (NLP), chatbots and generative language models, aimed at improving clinical training and supervision. Particular emphasis was placed on the use of these technologies for the analysis of therapeutic processes, the simulation of clinical interactions and the provision of structured feedback to foster competence and expertise in professionals in training.

#### 2.4.3 Context

The review was limited to practices of implementation and evaluation of these technologies in academic and clinical professional training settings. This context encompassed undergraduate courses, postgraduate programs, residencies, teaching clinics and other supervised or institutional settings aimed at training and developing skills in mental health.

#### 2.4.4 Types of Sources

This scoping review included studies containing experimental and quasi‐experimental designs (randomized controlled trials, non‐RCTs, before‐and‐after studies and interrupted time‐series studies), analytical observational studies (prospective and retrospective cohorts, case–control studies, analytical cross‐sectional studies) and descriptive observational designs (case series, individual case reports and descriptive cross‐sectional studies). Studies using quantitative, qualitative, or mixed‐methods approaches were considered. Grey literature included reports, theses, conference proceedings, guidelines, policies, websites and opinion pieces. Reference lists of identified scoping and systematic reviews were screened to identify further relevant studies.

Studies focused exclusively on patients without an interface with supervision or professional training were excluded to ensure methodological focus.

### 2.5 Search Strategy

The search strategy was outlined in three sequential steps as recommended by Peters et al. (2021). Preliminary research was conducted to support the introduction and justification of the protocol, drawing on the prior expertise of one author. On March 21, 2025, using the advanced search function, an initial search was carried out in Ovid/MEDLINE, as detailed in Supporting Information 1. This supplementary appendix provides the full Ovid/MEDLINE search strategy, including search terms, operators and record counts. As the screening process took longer than planned, an update was conducted on May 22, 2026, covering the period from March 21, 2025, to May 22, 2026, to identify additional eligible publications.

Subsequently, the strategy was adapted and replicated across eight additional databases (PsycINFO, EMBASE, LILACS, Web of Science, Scopus, ERIC, IEEE Xplore and ACM Digital Library) to ensure the retrieval of multidisciplinary evidence and methodological harmony across heterogeneous platforms.

Grey literature was considered an important source for scoping reviews, especially in quickly developing areas such as AI. Google Scholar was limited to the first 100 results. Reference lists of all included studies were screened using the snowball method. No language or time restrictions were applied. DeepL Translator and Google Translate were used to assess non‐English sources for eligibility; sources that could not be translated were excluded.

For eligible studies with incomplete data or methodological omissions, a protocol was established to contact corresponding authors via email. Articles whose authors did not respond within 10 business days were excluded to ensure the validity of the evidence synthesis.

### 2.6 Study Selection

After the search was completed, all identified articles were imported into the Rayyan software (Ouzzani et al. 2016) for organization and screening. A pilot test was conducted in which two reviewers evaluated 25 articles based on the eligibility criteria to guarantee consistency. Discrepancies had been discussed and the screening process continued once a minimum agreement level of 75% was achieved.

Following the pilot test, the remaining titles and abstracts were independently and blindly reviewed by two reviewers. Articles deemed potentially relevant were retrieved for full‐text assessment. Disagreements at any stage were resolved through discussion; when unresolved, a third reviewer made the final decision. The PRISMA‐ScR checklist (Tricco et al. 2018) guided the reporting process.

### 2.7 Data Charting

Data charting was performed using a structured instrument aligned with the PCC mnemonic and the review question. The instrument, based on JBI guidance for scoping reviews, was adapted to meet the specific requirements of the study. AI‐assisted preliminary extraction was performed in Rayyan (Ouzzani et al. 2016) only to support initial charting; each extracted field was verified and compared with the original source by human reviewers.

The data extraction protocol incorporated the following predefined categories: authors, year of publication, country of origin, study objective, population and sample size, study methodology, type of AI used, main findings and conclusions or recommendations.

### 2.8 Data Analysis and Presentation

Data analysis included frequency counts for descriptive quantitative synthesis and high‐level thematic categorization of AI applications, training contexts, outcomes and implementation issues. Results are presented using a PRISMA‐ScR flow diagram, a summary table and narrative synthesis aligned with the review objectives. Consistent with scoping review methodology, no formal critical appraisal was undertaken; therefore, findings are interpreted as a map of the evidence base rather than as conclusive evidence of effectiveness.

## 3 Results

### 3.1 Study Selection

Searches identified 11,975 records. After removal of 4381 duplicates, 7594 records were screened by title and abstract and 6777 were excluded. A total of 817 reports proceeded to full‐text assessment; 744 were excluded and 73 sources of evidence were included in the synthesis. Conference publication, dissertation status and review design were not exclusion criteria in themselves. Figure 1 presents the study selection process.

**FIGURE 1:**
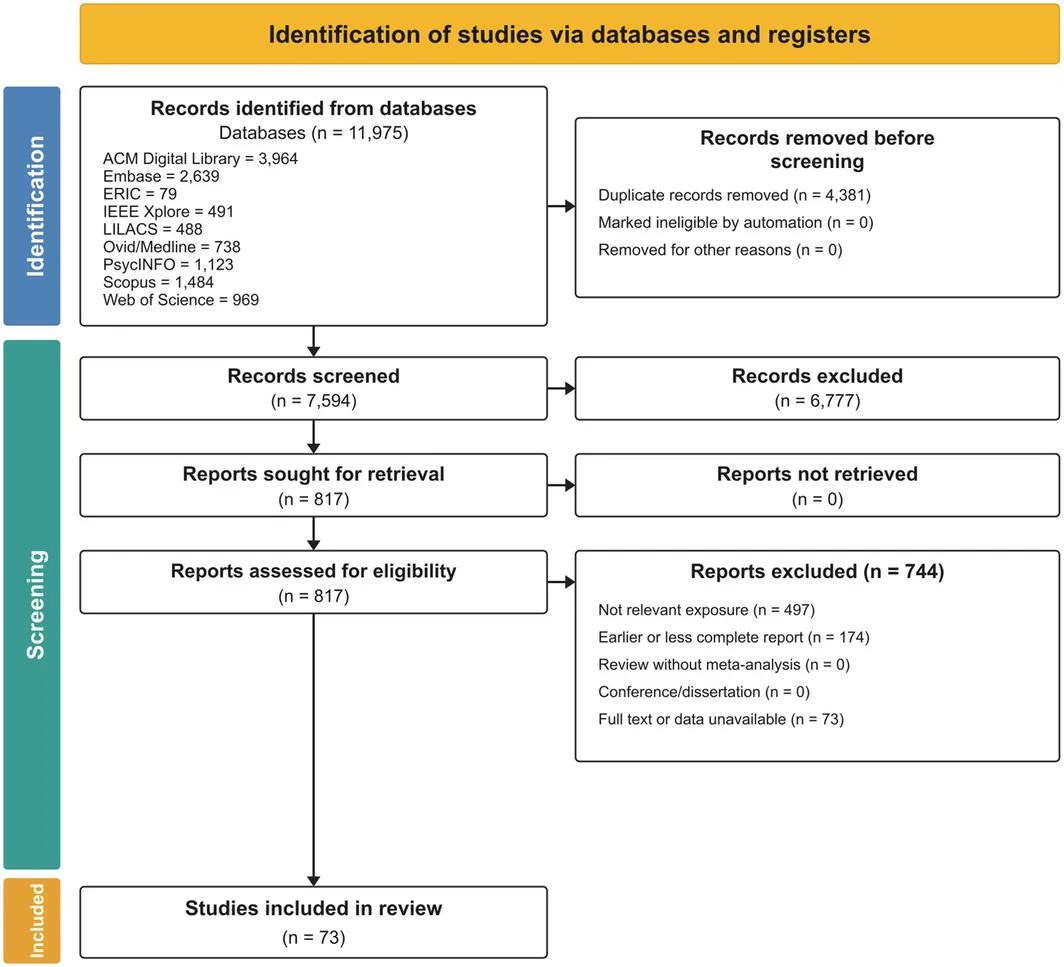
PRISMA‐ScR flow diagram of study selection.

### 3.2 Characteristics of Included Sources

The mapping included 73 sources of evidence relevant to AI applications in clinical supervision and training, spanning 2008 to 2026. The characteristics and full bibliographic details of all 73 included sources are provided in Supplementary Table S1. The table also identifies sources included in the descriptive mapping that are not discussed individually in the narrative synthesis. Publications were concentrated in recent years and encompassed empirical evaluations, methodological studies and other eligible sources. This range provides a broad account of research activity but should not be interpreted as 73 independent tests of training effectiveness.

Empirical designs included randomized comparisons, before‐and‐after evaluations, qualitative studies and computational validation. Protocols and reviews contributed information about planned research and the wider literature. Sample sizes and units of analysis varied, including trainees, other participants, sessions, utterances and datasets; these units should not be combined as if they represented equivalent training samples.

Technologies included GPT‐family LLMs, custom machine learning models and specialized clinical platforms such as Lyssn. Settings included university programs, residency training, community services and online training. Applications addressed CBT, motivational interviewing, general counselling skills, suicide prevention and multicultural orientation. Some evidence was indirect because the evaluated population, task, or dataset differed from routine supervision of mental health professionals. Table 1 summarizes the four application categories, predominant AI technologies and typical functions and settings.

**TABLE 1:** Summary of included studies by application category.

Application category	Predominant AI technology	Typical functions and settings
Virtual patient simulation	LLMs, chatbots, conversational agents, voice‐based avatars	Low‐stakes clinical role‐play, repeated skills practice, realistic client narratives and debriefing in university, residency and online training settings.
Automated performance assessment	Custom machine learning models, NLP classifiers, transformer models	Classification of therapist utterances, coding of discrete therapeutic skills, fidelity assessment, empathy ratings and multicultural‐orientation indicators.
AI‐enhanced supervisory feedback	LLMs and specialized clinical supervision platforms	Post‐session summaries, live or structured feedback, alternative clinical interpretations, technique recommendations and support for human supervision.
Training analytics and outcome monitoring	NLP, topic modelling, prediction models, working‐alliance analytics	Analysis of training/session data, outcome prediction, monitoring of therapeutic discourse and large‐scale quality improvement in training datasets.

*Note:* Categories are descriptive groupings derived from high‐level content categorization rather than mutually exclusive intervention classes.

Abbreviations: AI = artificial intelligence; LLM = large language model; NLP = natural language processing.

### 3.3 Reported Training and Clinical Outcomes

The reported outcomes addressed different questions and should be interpreted separately. Self‐efficacy and perceived improvement concern learners' appraisals; performance on a simulated task concerns behaviour in that task; algorithm validation concerns the quality of a scoring or classification system; and client outcomes concern clinical change. None can automatically substitute for another.

For behaviour‐based, automatically scored task performance, Tanana et al. (2019) randomized 151 nontherapist participants to conditions in a ClientBot study. The proportion of utterances classified as reflections was 91% higher with feedback and 76% higher after feedback was removed than in the comparison group. These are between‐group differences in a specific counselling behaviour, not percentage gains in overall competence; the scoring used automated classifiers. Young et al. (2026) reported greater improvement in AI‐scored CBT skills among trainees receiving AI feedback, using historical cohorts and analogue role‐plays. The nonrandomized design and use of AI for both feedback and assessment limit conclusions about independently assessed competence and transfer to clinical practice.

#### 3.3.1 Algorithm Validation and Diagnostic Recognition

Algorithm‐validation studies reported agreement with human ratings on selected tasks. Lubbe et al. (2026) reported a correlation of *R* = 0.60 for empathy ratings in a segment‐based assessment approach. Hammerfald et al. (2025) reported an F1 score of 0.78 and Cohen's κ of 0.69 for identifying microcounseling skills. These estimates describe scoring or classification performance within the study conditions; they do not demonstrate equivalence to the full role of a human supervisor or show that trainees improved.

In a feasibility study with AI‐simulated patients, medical students correctly identified depression severity in 60% and suicidality phase in 67% of cases (Holderried et al. 2026). These descriptive recognition outcomes do not establish improvement from baseline, appropriate treatment selection, or the ability to deliver effective treatment. Goldberg et al. (2025) reported correlations between machine‐generated and human ratings of multicultural orientation; these results concern measurement of recorded material rather than the development of trainees' cultural competence.

#### 3.3.2 Client Outcomes in Training Settings

Sheperis et al. (2025) described reductions in depression and anxiety symptoms in a university clinic using AI‐supported supervision. Such observations are clinically relevant, but do not isolate the contribution of AI from treatment, human supervision, or other features of the service. Kuo, Tanana, et al. (2024) analyzed the prediction of client distress from session recordings; this is evidence about outcome monitoring rather than a test of an AI training intervention. Patient improvement in a dataset should not be presented as evidence that an AI tool caused better care.

#### 3.3.3 Affective and Confidence Outcomes

Jeong et al. (2025) reported increases in counselling self‐efficacy and reductions in anxiety in a comparison of AI feedback with self‐review following chatbot practice. Although self‐efficacy trajectories differed, post hoc between‐group contrasts were not significant after multiplicity correction. Steenstra et al. (2025), Levkovich et al. (2025) and Elyoseph et al. (2025) also reported self‐efficacy outcomes in their respective training contexts. Sanz et al. (2025) measured negative affect and perceived knowledge and skills. These findings concern confidence, affect and perceived capability. They do not by themselves demonstrate acquisition of therapeutic skills. Grundmann et al. (2025) reported a decline in self‐efficacy after use of a conversational agent, indicating that confidence may change in either direction.

#### 3.3.4 Performance Feedback Quality

Feedback quality and perceived usefulness were assessed separately from trainee competence. Herschbach et al. (2025) reported that 85.38% of real‐time feedback was partially or totally correct; 83.64% of participants considered it accurate and 87.27% found it helpful. A judgement of partial correctness is not equivalent to consistently correct clinical guidance. Zech et al. (2022) evaluated prediction of human‐rated facilitative interpersonal skills, while Pellemans et al. (2024) evaluated automated coding of motivational interviewing behaviours. These studies support investigation of assessment tools but do not establish that receiving their scores improves treatment skills.

#### 3.3.5 Comparisons Between Training Formats

Comparative findings depended on the outcome assessed. Sawah et al. (2026) reported more favourable participant ratings for voice‐based avatars than text‐based chatbots, including perceived skill improvement. Cioffi et al. (2025) compared ratings of supervisory feedback generated under different conditions, including human feedback. Perceived improvement and favourable ratings of a feedback product should be distinguished from independent demonstrations of trainee skill development. These comparisons do not establish the general superiority of AI‐supported supervision.

### 3.4 Categories of AI Implementation

#### 3.4.1 Virtual Patient Simulation

Virtual patient simulation was a prominent application, enabling repeated practice through text or voice interactions. Studies examined the plausibility of generated client narratives, usability and stability of the simulated role (Tian et al. 2025; Cabrera Lozoya et al. 2025; Holderried et al. 2026). These are feasibility and experience outcomes; demonstrating that a simulation is usable or realistic is an initial step toward evaluating its educational value.

Systems varied in interactivity and realism. Text‐based chatbots and voice‐based avatars offered different experiences of practice (Sawah et al. 2026). The FASSLING approach combined role‐play with coaching and debriefing (Zhu 2025). Participants in other studies described overly agreeable responses, limited complexity and insufficient nonverbal information (Schmidt et al. 2025; Akkurt et al. 2025). Such limitations may constrain practice of challenging relational situations.

#### 3.4.2 Automated Performance Assessment and Feedback

Automated assessment applications classified therapeutic behaviours or estimated ratings from clinical material. Hammerfald et al. (2025) examined microcounseling skills and Lubbe et al. (2026) compared segment‐based and holistic assessment procedures. Agreement with human raters was task‐dependent. Standardized output should not be assumed to be unbiased or to capture the contextual judgement required in supervision.

Herschbach et al. (2025) examined use of specific communication techniques alongside real‐time feedback. Imel et al. (2024) evaluated detection of suicide risk assessment in crisis‐counselling calls, reporting different performance at call and statement levels. Detection of whether an assessment occurred is distinct from evaluating its adequacy, clinical decision‐making, or the effectiveness of the subsequent intervention.

#### 3.4.3 Supervisory Support and Professional Development

Applications also supported reflection on clinical material. Matthews et al. (2025) examined AI as a reflective partner and Sokolovskaya (2024) discussed supervisory uses such as generating alternative interpretations and resources. Hsieh et al. (2024) evaluated expert judgments of ChatGPT‐generated case conceptualizations. Mendlovic (2025) described AI‐assisted deliberate practice in psychodynamic psychotherapy. These reports show that higher‐order functions have received exploratory attention, but evaluation of generated content is different from evidence that trainees acquired these competencies.

AI‐supported clinic workflows included session summaries and monitoring of interventions, themes and symptom change (Sheperis et al. 2025). Peavy et al. (2024) discussed technology‐supported counselling and supervision in opioid treatment programs. Such functions may help supervisors select material for review; their clinical value depends on verification of the outputs and integration into human supervisory work.

#### 3.4.4 Training Data Analytics and Outcome Monitoring

Kuo, Tanana, et al. (2024) predicted next‐session symptom ratings from the linguistic content of the previous session (test‐set Spearman's *ρ* = 0.32, *p* < 0.001; *R* ^2^ = 0.07). These are predictive‐performance estimates rather than intervention effects. Topic modelling was also used to describe patterns in therapeutic discourse (Vanin et al. 2025).

The COMPASS framework used language modelling to examine patient‐therapist alliance strategies (Lin et al. 2024). Computational representations of an alliance provide potential material for investigation and feedback, but are not direct demonstrations of improved alliance management or training effectiveness.

#### 3.4.5 Ethical and Cultural Issues Across Applications

Ethical and cultural issues occurred across the four application categories. İçen and Köken (2025) examined AI‐generated guidance in ethically challenging child and adolescent psychiatry scenarios. Kuo, Mehta, et al. (2024) examined automated identification of cultural conversations in therapy. Producing guidance or recognizing cultural content is not equivalent to demonstrating that a trainee can reason ethically or respond appropriately to a client's cultural context.

Studies of simulated clients also raised questions about cultural representation and the effects of prompting (Akkurt et al. 2025; García‐Torres et al. 2025). These findings support attention to culturally responsive design and supervisory review of model outputs, rather than assuming that a plausible simulation adequately represents diverse clients.

### 3.5 Patterns Across Study Contexts

The following patterns provide context for interpreting the mapping. Because outcomes, populations and designs differ, they should be treated as descriptive observations and hypotheses for further study rather than tested moderators of effectiveness.

#### 3.5.1 Modality and Interaction Design

Interaction format influenced users' experience: avatar and text‐based simulations received different ratings (Sawah et al. 2026). Assessment procedure also mattered, with segment‐based and holistic prompting producing different agreement with human ratings (Lubbe et al. 2026). These findings address distinct outcomes and cannot be combined into a single estimate of educational effectiveness.

#### 3.5.2 Feedback Timing and Structure

Feedback during practice was associated with more reflections in the ClientBot experiment (Tanana et al. 2019). In contrast, the comparison of AI feedback and self‐review in Jeong et al. (2025) concerned self‐efficacy and anxiety. Together these examples indicate why the content and timing of feedback must be considered alongside the type of outcome, rather than assuming that favorable confidence scores demonstrate skill acquisition.

#### 3.5.3 Training Stage and Prior Experience

Participants ranged from people without therapist training to professional trainees and practicing counsellors. Tanana et al. (2019) studied nontherapists, whereas Young et al. (2026) studied psychotherapy trainees. Differences between these studies cannot isolate the effect of the training stage. Transfer to supervised clinical work requires direct evaluation in the relevant population.

#### 3.5.4 Domain‐Specific Considerations

CBT and motivational interviewing offered structured behaviors and fidelity frameworks for automated assessment (Creed et al. 2022; Steenstra et al. 2025). Their representation in the literature does not establish greater AI effectiveness in these modalities. In particular, the Project AFFECT publication is a study protocol, not a completed effectiveness trial (Creed et al. 2022). Relational and culturally responsive performance requires evaluation beyond the detection of protocol‐consistent utterances.

#### 3.5.5 Implementation Context and Support

Training applications were embedded in varied educational and service contexts (Kopelovich et al. 2025; Schmidt et al. 2025). Human supervision, curricular activities and practice opportunities can accompany AI use and may contribute to observed change. Comparisons across these settings do not isolate the added value of AI or establish that a hybrid model is superior without an appropriate comparison design.

#### 3.5.6 Quality and Sophistication of AI Systems

Ratings of AI‐generated content varied with model configuration, prompting and the task used for evaluation (Cioffi et al. 2025; Akkurt et al. 2025; Shusterman et al. 2025). A favourable rating of a generated response is evidence about that response under the tested conditions. It should not be generalized to the system's ability to supervise a clinician or produce durable improvements in care.

## 4 Discussion

### 4.1 Principal Findings

This scoping review mapped 73 sources of evidence relevant to AI applications in clinical supervision and training. The four overlapping categories describe uses of technology, rather than four established forms of effective supervision. The literature includes simulations, tools for coding clinical material, systems that generate feedback and analyses of training or clinical‐session data. The distinction between these functions is necessary because evidence that a tool can perform a task is not necessarily evidence that its use improves professional competence.

The adjunctive interpretation follows from the outcome‐specific reading of the evidence. Practice studies show that selected behaviours can change in a simulated task (Tanana et al. 2019); self‐efficacy studies address perceived capability (Jeong et al. 2025); and validation studies address agreement with human judgements (Lubbe et al. 2026). These findings identify potentially useful components of training but leave a gap between the evaluated task and broad clinical competence. Reports of client change do not close that gap when they cannot isolate the contribution of AI.

Accordingly, the recommendation to retain human supervision is an interpretive conclusion based on the limited scope of the demonstrated outcomes and the wider functions of supervision. It is not a claim that hybrid supervision has been shown to be superior in all settings. The same reasoning informs the research priorities: independent performance assessment is needed to evaluate competence, clinical follow‐up to evaluate transfer and direct study of relational supervisory processes to evaluate higher‐order functions.

### 4.2 Relationship to Prior Reviews

The present mapping complements reviews of technology‐based counselling training, machine learning in psychotherapy and AI in psychiatric and psychological education (Aafjes‐van Doorn et al. 2021; Magill et al. 2022; Lee et al. 2025; Prégent et al. 2025; Weightman et al. 2025). Its contribution is to consider these applications in relation to supervisory functions and the inferential distance between a reported outcome and competent clinical practice. This perspective allows feasibility, perceived benefit, algorithm performance and clinical learning to be discussed without treating them as interchangeable.

### 4.3 Implications for Clinical Training and Supervision

Training programs should specify the competency being practised, the evidence needed to assess it and how a supervisor will review AI‐generated feedback. A trainee can report feeling more capable while still needing support with formulation, intervention choice, timing and responsiveness to the client. Conversely, reduced confidence may reflect recognition of complexity rather than reduced competence. Neither interpretation should be accepted without observation of performance.

The distinction is supported by research on therapist self‐assessment outside the included AI literature. In a cognitive processing therapy implementation trial, self‐efficacy increased but was unrelated to independently rated adherence and competence (Pace et al. 2021). Brosan et al. (2008) found overestimation of competence, especially among less competent therapists, whereas McManus et al. (2012) found a tendency toward underestimation in trainees. The direction of error can vary; limited experience alone should not be treated as proof of overconfidence. Confidence remains a meaningful educational outcome, but it requires calibration against observed performance.

Higher‐order supervision includes helping trainees construct and revise case conceptualizations, manage anxiety and distress and examine transference, countertransference and the supervisory relationship (Borders 2014; American Psychological Association 2025). These functions connect discrete techniques to a particular client, therapist and therapeutic process. The clinical value of AI therefore depends partly on whether its outputs help human supervisors examine these processes.

For case conceptualization, an AI‐generated formulation may provide hypotheses for discussion. Hsieh et al. (2024) evaluated the acceptability of generated conceptualizations, which does not by itself show that trainees can integrate developmental history, context, maintaining processes and treatment priorities in their own work. Supervisory review should ask trainees to justify and revise a formulation using case evidence and to connect it to intervention choices. Identifying a disorder or risk state is only one element of this reasoning; it is not equivalent to treating the condition.

For anxiety and distress, the relevant question extends beyond whether a simulation makes trainees feel more comfortable. Supervision must help them recognize how distress affects attention, judgement, avoidance, or clinical boundaries. The anxiety outcomes reported by Jeong et al. (2025) concern training‐related affect and do not establish that an AI system can manage sustained distress or its effects on clinical practice. AI‐generated prompts may assist reflection, while the supervisor evaluates the trainee's needs and the implications for safe practice.

For transference and countertransference, AI may suggest questions about recurring relational patterns or identify material for joint review. Such suggestions remain hypotheses: the supervisor and trainee must examine the interaction, emotional responses, context and competing explanations. Exploratory reflective and psychodynamic applications (Matthews et al. 2025; Mendlovic 2025) do not establish that AI can reliably recognize or work through these processes. Future studies should assess formulation quality, responses to relational ruptures, reflective capacity and clinically appropriate use of countertransference information, alongside discrete technical skills.

### 4.4 Equity, Implementation and Governance

The concentration of studies in university‐affiliated and higher‐resource settings limits transferability to services with different languages, resources and training needs. Performance observed in a particular dataset or simulation may not generalize across cultures, professions, or clinical populations. Local evaluation should examine whose experiences are represented and whether a system's errors fall disproportionately on particular groups.

Implementation should therefore be guided by clinical governance rather than by technological availability alone. Training programs need explicit procedures for privacy, data security, bias monitoring, human review of AI‐generated feedback, trainee support and escalation when AI‐supported training reveals clinical risk or uncertainty.

### 4.5 Strength of the Evidence Base

No formal risk‐of‐bias appraisal was undertaken. The following qualifications therefore describe the design and measurement issues visible in the mapping, rather than a graded assessment of evidence certainty. Small or short‐term studies, nonrandomized comparisons and self‐reported outcomes limit causal interpretation. Independent ratings, blinded assessment where feasible and follow‐up in actual clinical practice, are needed to assess durable skill acquisition. If a system both trains and scores the trainee, improvement may partly reflect alignment with that system's scoring criteria; external validation is essential. Clinical outcome analyses also need to account for human supervision, treatment exposure, case mix and clustering of clients within therapists.

### 4.6 Limitations

This review has limitations at the level of the review process and the evidence mapped. First, it was designed to describe a heterogeneous literature and no formal critical appraisal or meta‐analysis was conducted. Application categories can overlap and narrative examples cannot establish pooled effectiveness, rank competing systems, or determine which training contexts benefit most. The interpretive distinctions between outcome types improve clarity but do not remove biases in the underlying studies.

Second, search and access decisions may have affected coverage. Restricting Google Scholar screening to the first 100 results, excluding sources that could not be translated and excluding reports with unresolved missing information may introduce retrieval or selection bias. AI‐assisted preliminary extraction in Rayyan also requires careful human verification because generated summaries can misclassify designs or outcomes. Rapid changes in models and their availability further limit the stability of a mapping of this field.

Third, short follow‐up, small or setting‐specific samples, self‐reported outcomes and limited independent assessment constrain inferences about competence and transfer. Evidence from simulations, algorithm‐validation datasets and groups outside routine mental health professional training is indirect for clinical supervision. Where AI both supplies feedback and assesses performance, shared scoring criteria may inflate apparent learning. Confidence, diagnostic recognition and ratings of generated content do not establish effective treatment delivery. Finally, client outcomes and higher‐order supervisory processes receive insufficient direct evaluation to support conclusions about patient benefit or replacement of human supervision. These limitations require cautious interpretation and define priorities for further research.

## 5 Conclusion

This scoping review maps AI applications in clinical supervision and training across virtual patient simulation, automated assessment, supervisory feedback and training analytics. The evidence supports further investigation of these tools as adjuncts for selected learning and assessment tasks. It does not establish that increased self‐efficacy, successful diagnostic recognition, or agreement between AI and human ratings translates into broader treatment competence. Case conceptualization, management of supervisee anxiety and distress and exploration of transference and countertransference remain central to supervision and require explicit evaluation in AI‐supported models. Conclusions about client benefit and replacement of human supervisory functions are premature. Future research should combine independently assessed competence with clinical follow‐up, transparent model reporting and attention to cultural and service contexts. Overall, the findings support investigating AI as an adjunct to, rather than a replacement for, human clinical supervision. Its contribution should ultimately be judged not by the sophistication or efficiency of the technology alone, but by its demonstrated capacity to support the development of competent practitioners and the delivery of safe, effective and equitable mental health care.

## Funding

The authors have nothing to report.

## Conflicts of Interest

The authors declare no conflicts of interest.

## Supporting information




**Supplementary Table S1.** Characteristics and bibliographic details of the 73 sources included in the scoping review.

**Supporting Information 1.** Full Ovid/MEDLINE search strategy for the initial search conducted on March 21, 2025, including search terms, operators and record counts.

## Data Availability

The extracted data, search strategies, and supplementary tables will be made available as Supporting Information and/or in an open repository after removal of any identifying information required for peer review.
