# Supplementary material for: Artificial Intelligence in Clinical Supervision and Training of Psychologists and Other Mental Health Professionals: A Scoping Review
Source: Clin Psychol Psychother. 2026 Nov 9;33(6):e70359. doi: 10.1002/cpp.70359 (PMC13748793; doi:10.1002/cpp.70359)
Supplement: Supplementary file 1 [file CPP-33-e70359-s001.docx]

Supplementary Table S1. Characteristics and full bibliographic details of the 73 sources included in the scoping review.

| **#** | **Study** | **Country** | **Study type** | **Setting** | **Domain** | **AI technology** | **Model access** | **Application category** | **Supervisory focus** | **Outcome level** |
| --- | --- | --- | --- | --- | --- | --- | --- | --- | --- | --- |
| 1 | Sanz et al. (2025) | Spain | Intervention | Private university undergraduate program | Communication skills development | ChatGPT-3.5 | Proprietary | Virtual patient simulation | Intervention | 2 |
| 2 | Xu et al. (2025) | China | Computational | Counselling-psychology graduate program | Therapeutic skill practice | Llama3.1-8B, Qwen3-8B/14B, GPT-4o, Claude-Sonnet-4 | Both | Supervisory feedback | Intervention | 0 |
| 3 | Sawah et al. (2026) | United Kingdom | Intervention | Postgraduate clinical psychology | Cognitive-behavioural therapy | GPT-5 Model (chatbot and avatar) | Proprietary | Virtual patient simulation | Intervention | 2 |
| 4 | Jeong et al. (2025) | South Korea | Intervention | Master's program in counseling | Self-efficacy and anxiety reduction | ChatGPT-4o | Proprietary | Supervisory feedback | Intervention | 2 |
| 5 | Lubbe et al. (2026) | Germany | Computational | Brandenburg Medical School | Psychotherapeutic communication | ChatGPT version o1 | Proprietary | Automated assessment | Intervention | 0 |
| 6 | Creed et al. (2022) | USA | No empirical data | Community mental health agencies | Cognitive Behavioral Therapy | LyssnCBT platform | Commercial platform | Supervisory feedback | Intervention | — |
| 7 | Steenstra et al. (2025) | USA | Intervention | Professional and student counselor training | Motivational interviewing | GPT-4o | Proprietary | Virtual patient simulation | Intervention | 2 |
| 8 | Grundmann et al. (2025) | Netherlands | Intervention | Dutch child helpline | Five Phase Model counseling | Custom BDI model | Custom model | Virtual patient simulation | Intervention | 2 |
| 9 | Lushyn & Sukhenko (2025) | Ukraine | Intervention | Diverse educational backgrounds | Eco-Centered Psychological Facilitation | Claude 3.5 Sonnet | Proprietary | Supervisory feedback | Intervention | 2 |
| 10 | Cabrera Lozoya et al. (2025) | Australia | Qualitative or user perception | Academic environment | Depression and anxiety (CBT) | GPT-4 | Proprietary | Virtual patient simulation | Intervention | 1 |
| 11 | Cioffi et al. (2025) | Italy | Computational | Psychotherapy training | Gestalt psychotherapy | ChatGPT-4 | Proprietary | Supervisory feedback | Intervention and conceptualization | 0 |
| 12 | Ranihusna (2025) | Indonesia | Intervention | Clinical training program | Tele-mental health counseling | AI-enhanced feedback system | Not reported | Supervisory feedback | Intervention | 3 |
| 13 | Young et al. (2026) | USA | Intervention | Doctoral clinical psychology program | Cognitive Behavioral Therapy | Lyssn.io Quality Improvement tool | Commercial platform | Supervisory feedback | Intervention | 3 |
| 14 | Kopelovich et al. (2025) | USA | Intervention | Community behavioral health agency | CBT for psychosis | Custom CBT training tool with AI feedback | Custom model | Supervisory feedback | Intervention | 1 |
| 15 | Tanana et al. (2019) | USA | Intervention | Web-based system (Amazon MTurk) | Basic counseling skills | ClientBot (neural conversational agent) | Custom model | Virtual patient simulation | Intervention | 3 |
| 16 | Holderried et al. (2026) | Germany | Intervention | Medical school | Depression assessment and suicidality | GPT-4 | Proprietary | Virtual patient simulation | Intervention | 3 |
| 17 | Schmidt et al. (2025) | Australia | Qualitative or user perception | Masters psychology students | CBT, ACT, DBT | GPT-4 (Client101) | Proprietary | Virtual patient simulation | Intervention | 1 |
| 18 | Herschbach et al. (2025) | Germany | Intervention | University Hospital training | Mental health communication | GPT-4 | Proprietary | Virtual patient simulation | Intervention | 3 |
| 19 | DeMasi et al. (2020) | USA | Qualitative or user perception | Training program for mental health professionals | Crisis hotline counseling | Custom chatbot with pre-trained model | Custom model | Virtual patient simulation | Intervention | 1 |
| 20 | Akkurt et al. (2025) | USA | Qualitative or user perception | Online counseling program | General counseling skills | ChatGPT GPT-4o | Proprietary | Virtual patient simulation | Intervention | 1 |
| 21 | Sri et al. (2025) | India | Computational | Not specified | Mental health scenario simulation | Explainable GANs | Custom model | Virtual patient simulation | Intervention | 0 |
| 22 | Georgila et al. (2019) | USA | Intervention | U.S. Army officer training | Basic counseling skills | Reinforcement Learning ITS | Custom model | Virtual patient simulation | Intervention | 2 |
| 23 | Goldberg et al. (2025) | USA | Computational | Graduate psychology programs | Multicultural orientation | RoBERTa transformer model | Custom model | Automated assessment | Intervention | 0 |
| 24 | García-Torres et al. (2025) | Spain | Intervention | Miguel Hernández University | Psychopathology | ChatGPT gpt-4o model | Proprietary | Virtual patient simulation | Intervention | 2 |
| 25 | Hsieh et al. (2024) | Taiwan | Computational | Academic environment | Case conceptualization | ChatGPT | Proprietary | Supervisory feedback | Conceptualization | 0 |
| 26 | Sheperis et al. (2025) | USA | Intervention | University telemental health clinic | General counseling | Eleos Health platform | Commercial platform | Supervisory feedback | Intervention | 5 |
| 27 | Fung & Laing (2024) | Not determined | Computational | Healthcare educational settings | CBT for depression | ChatGPT 4 | Proprietary | Virtual patient simulation | Intervention | 0 |
| 28 | Hirsch et al. (2018) | USA | Qualitative or user perception | Clinical training programs | Motivational Interviewing | CORE-MI (machine learning) | Custom model | Automated assessment | Intervention | 1 |
| 29 | Tian et al. (2025) | USA | Computational | Counselor education programs | General counseling skills | GPT-4 | Proprietary | Virtual patient simulation | Intervention | 0 |
| 30 | Hammerfald et al. (2025) | Not determined | Computational | Psychology training | Microcounseling skills | GPT-4.1 | Proprietary | Automated assessment | Intervention | 0 |
| 31 | Sokolovskaya (2024) | Not determined | Qualitative or user perception | Mental health professionals | Positive and Transcultural Psychotherapy | SupervizAI (text-based neural network) | Not reported | Supervisory feedback | Conceptualization | 1 |
| 32 | Levkovich et al. (2025) | Israel | Intervention | Online webinar | Suicide prevention (QPR) | GPT-4o (OpenAI) | Proprietary | Virtual patient simulation | Intervention | 2 |
| 33 | Elyoseph et al. (2025) | Israel | Intervention | Live webinar | Suicide risk assessment (QPR) | GPT-4o (OpenAI) | Proprietary | Virtual patient simulation | Intervention | 2 |
| 34 | Lockwood et al. (2025) | USA | Computational | School psychology programs | Qualitative research methods | ChatGPT-4 | Proprietary | Adjacent application | — | 0 |
| 35 | Q. Y. Lee et al. (2025) | Singapore | Review | Medical education | Psychiatric education | ChatGPT, Claude 3, Llama 3 | — | — | — | — |
| 36 | Donnelly et al. (2026) | USA | Intervention | Virtual workshop | Suicide prevention research | Jupyter notebooks with Azure Databricks | — | Adjacent application | — | 2 |
| 37 | Yosef et al. (2024) | Israel | Computational | Not specified | Motivational interviewing | GPT-3.5-turbo-1106 | Proprietary | Virtual patient simulation | Intervention | 0 |
| 38 | Y. Zhu (2025) | Not determined | No empirical data | Clinical psychology training | General therapeutic skills | FASSLING platform | Not reported | Virtual patient simulation | Intervention | — |
| 39 | Zech et al. (2022) | USA | Computational | Messaging therapy training | Facilitative interpersonal skills | DistilBERT | Custom model | Automated assessment | Intervention | 0 |
| 40 | Peavy et al. (2024) | USA | Qualitative or user perception | Opioid treatment program | Motivational interviewing | Lyssn.io | Commercial platform | Automated assessment | Intervention | 1 |
| 41 | Benamara et al. (2023) | France | Qualitative or user perception | Healthcare training | Emotional disorders (Alzheimer's) | COPALZ model (BDI-based) | Custom model | Virtual patient simulation | Intervention | 1 |
| 42 | Han et al. (2024) | USA | Computational | University students | Motivational interviewing | Llama2-13B-Chat, GPT models | Both | Automated assessment | Intervention | 0 |
| 43 | Al Owayyed et al. (2024) | Netherlands | Qualitative or user perception | Child helpline training | Five Phase Model | Custom BDI model (Lilobot) | Custom model | Virtual patient simulation | Intervention | 1 |
| 44 | Aafjes-van Doorn et al. (2025) | USA | Computational | Clinical psychology training | General therapeutic skills | Multilayer perceptron | Custom model | Automated assessment | Intervention | 0 |
| 45 | Mendlovic (2025) | Israel | No empirical data | Likely residency/continuing education | Psychodynamic psychotherapy | ChatGPT-4 (OpenAI) | Proprietary | Supervisory feedback | Conceptualization | — |
| 46 | Shusterman et al. (2025) | USA | Computational | Professional training | CBT for Insomnia | Meta LLaMa 2 | Open | Adjacent application | — | 0 |
| 47 | Moran et al. (2025) | USA | Qualitative or user perception | Not applicable | Cognitive Behavioral Therapy | Hypothetical AI teammate | — | Supervisory feedback | Intervention | 1 |
| 48 | Napiwotzki et al. (2025) | Germany | Computational | Medical school/residency | Behavioral activation therapy | GPT-4, GPT-4o, Gemini, Claude, Llama-3, Command R+ | Both | Adjacent application | — | 0 |
| 49 | Orrù et al. (2024) | Italy | Computational | Not specified | Clinical question analysis | BERT-based ML model (MADIT) | Custom model | Training analytics | Intervention | 0 |
| 50 | Pellemans et al. (2024) | Netherlands | Computational | Online suicide prevention helpline | Motivational Interviewing | BERTje (Dutch BERT) | Custom model | Automated assessment | Intervention | 0 |
| 51 | X. T. Zhu et al. (2025) | Not determined | Qualitative or user perception | Health professions education | Clinical communication | GPT-4o with ECAs | Proprietary | Virtual patient simulation | Intervention | 1 |
| 52 | Guhan et al. (2025) | USA | Computational | Medical schools | Patient engagement assessment | Custom multimodal semisupervised GAN | Custom model | Adjacent application | — | 0 |
| 53 | Gibson et al. (2016) | USA | Computational | Not mentioned | Empathy in motivational interviewing | Deep neural network (RNN) | Custom model | Automated assessment | Intervention | 0 |
| 54 | Matthews et al. (2025) | Not determined | Qualitative or user perception | Not specified | Reflective practice | GAI clinical note-taking platforms | Commercial platform | Supervisory feedback | Conceptualization | 1 |
| 55 | Sheperis & Sadeh-Sharvit (2023) | USA | Qualitative or user perception | University telemental health clinic | Anxiety and depression assessment | Machine learning model | Commercial platform | Supervisory feedback | Intervention | 1 |
| 56 | Vanin et al. (2025) | Russia | Computational | Not mentioned | Humanistic and CBT approaches | BERTopic with Sentence-Transformer | Custom model | Training analytics | Intervention | 0 |
| 57 | Creed et al. (2021) | USA | Qualitative or user perception | Community mental health care | CBT fidelity | CORE-MI adapted for CBT | Custom model | Automated assessment | Intervention | 1 |
| 58 | Wang et al. (2025) | China | Review | Academic and clinical environments | Psychotherapeutic techniques | Intelligent Tutoring Systems | — | — | — | — |
| 59 | Cruz et al. (2025) | Mexico | Qualitative or user perception | Addiction Prevention Center, UNAM | Depression, anxiety, substance abuse | PEPE (chatbot with ChatGPT-4o) | Proprietary | Virtual patient simulation | Intervention | 1 |
| 60 | Held et al. (2025) | USA | Qualitative or user perception | Postgraduate level | Cognitive restructuring (CBT) | Socrates 2.0 (multi-agent AI) | Not reported | Virtual patient simulation | Intervention | 1 |
| 61 | Gill & Galletly (2025) | Australia | No empirical data | RANZCP Fellowship Training | Psychiatry training | ChatGPT | Proprietary | — | — | — |
| 62 | Kuo, Mehta, et al. (2024) | USA | Computational | Clinical training programs | Cultural competency | SVM and RoBERTa | Custom model | Automated assessment | Intervention | 0 |
| 63 | Lin (2022) | USA | Computational | Not mentioned | Real-time treatment strategies | R2D2 (reinforcement learning) | Custom model | Supervisory feedback | Intervention | 0 |
| 64 | Lin et al. (2023) | USA | Computational | Medical school/residency | Anxiety, depression, schizophrenia, suicidality | DISMOP (Deep RL) | Custom model | Supervisory feedback | Intervention | 0 |
| 65 | Wu et al. (2022) | Not determined | Computational | Not mentioned | Therapist action forecasting | Language models | Custom model | Training analytics | Intervention | 0 |
| 66 | Imel et al. (2024) | USA | Computational | Crisis counseling training | Suicide risk assessment | RoBERTa transformer model | Custom model | Automated assessment | Intervention | 0 |
| 67 | Lin et al. (2024) | USA | Computational | Medical school/residency | CBT, depression, anxiety, schizophrenia | Doc2Vec, SentenceBERT, ChatGPT | Proprietary | Training analytics | Intervention | 0 |
| 68 | Masri et al. (2008) | Malaysia | No empirical data | Not specified | General mental health diagnostics | Expert System (rule-based AI) | Custom model | Adjacent application | — | — |
| 69 | Longobardi et al. (2018) | Italy | Computational | Not mentioned | Anger management | Machine learning emotion classifier | Custom model | Training analytics | Intervention | 0 |
| 70 | İçen & Köken (2025) | Türkiye | Computational | Not mentioned | Child and adolescent psychiatry ethics | ChatGPT 4.0, Gemini 2.5, GROK 3 | Proprietary | Supervisory feedback | Conceptualization | 0 |
| 71 | Magill et al. (2022) | USA | Review | Health and mental health training | CBT, motivational interviewing | Various (Lyssn, avatar simulations) | — | — | — | — |
| 72 | Abdou et al. (2025) | USA | Computational | Digital therapy service | Psychological distancing, CBT | LLaMA (Meta AI) version 3.1 | Open | Training analytics | Intervention | 0 |
| 73 | Kuo, Tanana, et al. (2024) | USA | Computational | Not specified | General therapeutic processes | RoBERTa | Custom model | Training analytics | Intervention | 0 |

Note. Study type: computational = development or validation of an AI tool without trainee participants; qualitative or user perception = opinions, usability, or experiences of users; intervention = participants used the tool and an outcome was measured. Outcome level (highest measured): 0 = technical performance of the AI tool; 1 = reactions and acceptability; 2 = self-assessed learning; 3 = observed skill in simulated or standardized tasks; 4 = skill or fidelity in real clinical sessions; 5 = client outcomes; — = no empirical outcome. Supervisory focus follows the discrimination model (Bernard & Goodyear, 2019). Setting, domain, and AI technology are reproduced from the data charting table.

# References for the 73 Included Sources

**01.** Sanz, A., Tapia, J. L., García-Carpintero, E., et al. (2025). ChatGPT Simulated Patient: Use in Clinical Training in Psychology. Psicothema. https://doi.org/10.70478/psicothema.2025.37.21

**02.** Xu, C., Lv, Z., Lan, T., et al. (2025). LLM-as-a-Supervisor: Mistaken Therapeutic Behaviors Trigger Targeted Supervisory Feedback. arXiv. https://doi.org/10.48550/arXiv.2508.09042

**03.** Sawah, V. E., Bhardwaj, A., Pryke-Hobbes, A., et al. (2026). Artificial Intelligence as a Training Tool in Clinical Psychology: A Comparison of Text-Based and Avatar Simulations. arXiv. https://doi.org/10.48550/arXiv.2601.11533

**04.** Jeong, D., Lee, H., Yang, H., et al. (2025). AI Integration in Counseling Training: Aiding Counselors-in-Training in Self-Efficacy Enhancement and Anxiety Reduction. Journal of Counseling and Development. https://doi.org/10.1002/jcad.12560

**05.** Lubbe, D., Wedderhoff, N., & Nelles, C. (2026). AI-driven versus human evaluations of psychotherapeutic communication performance: The impact of procedural prompting on assessment reliability and validity. Computers in Human Behavior Reports, 21, Article 100910. https://doi.org/10.1016/j.chbr.2025.100910

**06.** Creed, T. A., Salama, L., Slevin, R. A., et al. (2022). Enhancing the quality of cognitive behavioral therapy in community mental health through artificial intelligence generated fidelity feedback (Project AFFECT): a study protocol. BMC Health Services Research. https://doi.org/10.1186/s12913-022-08519-9

**07.** Steenstra, I., Nouraei, F., & Bickmore, T. W. (2025). Scaffolding Empathy: Training Counselors with Simulated Patients and Utterance-level Performance Visualizations. CHI Conference on Human Factors in Computing Systems. https://doi.org/10.1145/3706598.3714014

**08.** Grundmann, S., Al Owayyed, M., Bruijnes, M., et al. (2025). Lilobot: A Cognitive Conversational Agent to Train Counsellors at Children's Helplines: Design and Initial Evaluation. Journal of Medical Systems. https://doi.org/10.1007/s10916-024-02121-8

**09.** Lushyn, P., & Sukhenko, Y. (2025). Beyond Traditional Training: AI as a Supportive Instrument in Psychologist Development. Information Technologies and Learning Tools. https://doi.org/10.33407/itlt.v109i5.6160

**10.** Cabrera Lozoya, D., Conway, M., Sebastiano De Duro, E., & D'Alfonso, S. (2025). Leveraging large language models for simulated psychotherapy client interactions: Development and usability study of Client101. JMIR Medical Education, 11, e68056. https://doi.org/10.2196/68056

**11.** Cioffi, V., Ragozzino, O., Mosca, L. L., et al. (2025). Can AI Technologies Support Clinical Supervision? Assessing the Potential of ChatGPT. Informatics. https://doi.org/10.3390/informatics12010029

**12.** Ranihusna, D. (2025). The Virtual Supervision Revolution: Enhancing Counselor Competency in Tele-Mental Health Through AI-Enhanced Feedback Systems. International Journal of Research in Counseling. https://doi.org/10.70363/ijrc.v4i1.277

**13.** Young, J., Schruff, M. C., Mudryk, E., et al. (2026). Artificial Intelligence-generated Feedback as a Training Tool for Enhancing Psychotherapy Skill. Journal of Technology in Behavioral Science. https://doi.org/10.1007/s41347-026-00603-9

**14.** Kopelovich, S. L., Slevin, R. A., Brian, R., et al. (2025). Preliminary investigation of an artificial intelligence-based cognitive behavioral therapy training tool. Psychotherapy. https://doi.org/10.1037/pst0000550

**15.** Tanana, M. J., Soma, C. S., Srikumar, V., et al. (2019). Development and Evaluation of ClientBot: Patient-Like Conversational Agent to Train Basic Counseling Skills. Journal of Medical Internet Research. https://doi.org/10.2196/12529

**16.** Holderried, F., Sonanini, A., Philipps, A., et al. (2026). Using AI to Train Future Clinicians in Depression Assessment: Feasibility Study. JMIR Medical Education. https://doi.org/10.2196/87102

**17.** Schmidt, S., Lozoya, D. C., Kiropoulos, L., et al. (2025). Psychology student and mental health practitioner experiences of and perspectives on Client101, a virtual client chatbot training tool. BMC Medical Education. https://doi.org/10.1186/s12909-025-07668-9

**18.** Herschbach, L., Festl-Wietek, T., Stegemann-Philipps, C., et al. (2025). Evaluation of an AI-Based Chatbot Providing Real-Time Feedback in Communication Training for Mental Health Care Professionals: Proof-of-Concept Observational Study. Journal of Medical Internet Research. https://doi.org/10.2196/82818

**19.** DeMasi, O., Li, Y., & Yu, Z. (2020). A Multi-Persona Chatbot for Hotline Counselor Training. Findings of the Association for Computational Linguistics: EMNLP 2020.

**20.** Akkurt, M., Maurya, R., & Brown, T. (2025). Learning through simulation: Counselor trainees’ interactions with ChatGPT as a client. Behavioral Sciences, 15(12), Article 1660. https://doi.org/10.3390/bs15121660

**21.** Sri, L. P., Shri, R. R., Thrisha, R., & S, V. (2025). MindMend: Explainable GANs for Stimulating Mental Health Scenarios in Therapist Training. 2025 International Conference on Electronics and Renewable Systems (ICEARS). https://doi.org/10.1109/ICEARS64219.2025.10940062

**22.** Georgila, K., Core, M. G., Nye, B. D., et al. (2019). Using Reinforcement Learning to Optimize the Policies of an Intelligent Tutoring System for Interpersonal Skills Training. Proceedings of the 18th International Conference on Autonomous Agents and MultiAgent Systems.

**23.** Goldberg, S. B., Tanana, M. J., Stewart, S. H., et al. (2025). Automating the assessment of multicultural orientation through machine learning and natural language processing. Psychotherapy. https://doi.org/10.1037/pst0000519

**24.** García-Torres, D., Fernández, C., Mira, J. J., et al. (2025). PsicoSimGPT: Evaluating the Use of Generative AI for Training in Psychopathological Interviewing. https://doi.org/10.2196/preprints.78857

**25.** Hsieh, L.-H., Liao, W.-H., & Liu, E.-Y. (2024). Feasibility Assessment of Using ChatGPT for Training Case Conceptualization Skills in Psychological Counseling. Computers in Human Behavior: Artificial Humans. https://doi.org/10.1016/j.chbah.2024.100083

**26.** Sheperis, D., Sadeh-Sharvit, S., Guinn, V., Joseph, R., Bunge, E., & Assenheim-Dagan, L. (2025). Integrating artificial intelligence in a university counseling clinic. Journal of Counselor Preparation and Supervision, 19(3), 1–13. https://doi.org/10.70013/wdo9cs4m

**27.** Fung, L., & Laing, R. (2024). A proof of concept study on the use of large language models as a client in typed role plays for training therapists. Discover Psychology. https://doi.org/10.1007/s44202-024-00302-7

**28.** Hirsch, T., Soma, C. S., Merced, K., et al. (2018). "It's hard to argue with a computer": Investigating Psychotherapists' Attitudes towards Automated Evaluation. Designing Interactive Systems. https://doi.org/10.1145/3196709.3196776

**29.** Tian, X., Liu, Y., Litt, S., et al. (2025). Do Artificial Intelligence Clients Speak Like Human Clients? Exploring GPT-4's Content-Level Performance in Counseling Role-Play. Journal of Counseling and Development. https://doi.org/10.1002/jcad.70016

**30.** Hammerfald, K., Schmidt, S., Vlassov, V., et al. (2025). Leveraging large language models to identify microcounseling skills in psychotherapy transcripts. Psychotherapy Research. https://doi.org/10.1080/10503307.2025.2539405

**31.** Sokolovskaya, M. (2024). Enhancing supervisory practice in Positive and Transcultural Psychotherapy through Artificial Intelligence. The Global Psychotherapist. https://doi.org/10.52982/lkj253

**32.** Levkovich, I., Haber, Y., Levi-Belz, Y., & Elyoseph, Z. (2025). A step toward the future? Evaluating GenAI QPR simulation training for mental health gatekeepers. Frontiers in Medicine. https://doi.org/10.3389/fmed.2025.1599900

**33.** Elyoseph, Z., Levkovitch, I., Haber, Y., & Levi-Belz, Y. (2025). Using GenAI to Train Mental Health Professionals in Suicide Risk Assessment: Preliminary Findings. Journal of Clinical Psychiatry, 86(3), 24m15525. https://doi.org/10.4088/JCP.24m15525

**34.** Lockwood, A. B., Newman, D. S., Mossing, K. W., et al. (2025). Human versus machine: A comparative analysis of qualitative coding by humans and ChatGPT-4. School Psychology. https://doi.org/10.1037/spq0000715

**35.** Lee, Q. Y., Chen, M., Ong, C. W., & Ho, C. S. H. (2025). The role of generative artificial intelligence in psychiatric education: A scoping review. BMC Medical Education, 25, Article 438. https://doi.org/10.1186/s12909-025-07026-9

**36.** Donnelly, H., Mandell, D., Hwang, S., et al. (2026). Data Science Education for Residents, Researchers, and Students in Psychiatry and Psychology: Program Development and Evaluation Study. JMIR Medical Education. https://doi.org/10.2196/75125

**37.** Yosef, S., Zisquit, M., Cohen, B., et al. (2024). Assessing Motivational Interviewing Sessions with AI-Generated Patient Simulations. CLPsych 2024 - 9th Workshop on Computational Linguistics and Clinical Psychology.

**38.** Zhu, Y. (2025). Revolutionizing simulation-based clinical training with AI: Integrating FASSLING for enhanced emotional intelligence and therapeutic competency in clinical psychology education. Journal of Clinical Technology and Theory, 2, 38–54. https://doi.org/10.54254/3049-5458/2025.21247

**39.** Zech, J. M., Steele, R., Foley, V. K., & Hull, T. D. (2022). Automatic rating of therapist facilitative interpersonal skills in text: A natural language processing application. Frontiers in Digital Health. https://doi.org/10.3389/fdgth.2022.917918

**40.** Peavy, K. M., Klipsch, A., Soma, C. S., et al. (2024). Improving the quality of counseling and clinical supervision in opioid treatment programs: how can technology help? Addiction Science & Clinical Practice. https://doi.org/10.1186/s13722-024-00435-z

**41.** Benamara, A., Martin, J.-C., Prigent, E., & Ravenet, B. (2023). Evaluating a Model of Pathological Affect based on Pedagogical Situations for a Virtual Patient. Proceedings of the 23rd ACM International Conference on Intelligent Virtual Agents. https://doi.org/10.1145/3570945.3607325

**42.** Han, G., Liu, W., Huang, X., & Borsari, B. (2024). Chain-of-Interaction: Enhancing Large Language Models for Psychiatric Behavior Understanding by Dyadic Contexts. 2024 IEEE 12th International Conference on Healthcare Informatics (ICHI). https://doi.org/10.1109/ICHI61247.2024.00057

**43.** Al Owayyed, M., Despan, A., Tielman, M., & Brinkman, W.-P. (2024). A Cognitive Conversational Agent for Training Child Helpline Volunteers. Proceedings of the 24th ACM International Conference on Intelligent Virtual Agents. https://doi.org/10.1145/3652988.3696197

**44.** Aafjes-van Doorn, K., Cicconet, M., Bate, J., et al. (2025). Development of an artificial intelligence-based measure of therapists' skills: A multimodal proof of concept. Psychotherapy. https://doi.org/10.1037/pst0000561

**45.** Mendlovic, S. (2025). Using an AI-Based System for Dimensional Deliberate Practice in Psychodynamic Psychotherapy. Broad Research in Artificial Intelligence and Neuroscience. https://doi.org/10.70594/brain/16.3/34

**46.** Shusterman, R., Waters, A. C., O'Neill, S., et al. (2025). An active inference strategy for prompting reliable responses from large language models in medical practice. npj Digital Medicine. https://doi.org/10.1038/s41746-025-01516-2

**47.** Moran, L. H., Kee, S. C., Wiese, C. W., et al. (2025). Artificial Intelligence as a Feedback Teammate for Treatment Delivery: Cognitive Behavioral Therapists' Hopes and Fears. Cognitive and Behavioral Practice. https://doi.org/10.1016/j.cbpra.2025.06.007

**48.** Napiwotzki, I., Laue, J., Caldarone, F., et al. (2025). Comparing Human and AI Therapists in Behavioral Activation for Depression: Cross-Sectional Questionnaire Study. JMIR Formative Research. https://doi.org/10.2196/78138

**49.** Orrù, L., Cuccarini, M., Moro, C., & Turchi, G. (2024). Clinical Questions and Psychological Change: How Can Artificial Intelligence Support Mental Health Practitioners? Behavioral Sciences. https://doi.org/10.3390/bs14121225

**50.** Pellemans, M., Salmi, S., Merelle, S., et al. (2024). Automated Behavioral Coding to Enhance the Effectiveness of Motivational Interviewing in a Chat-Based Suicide Prevention Helpline: Secondary Analysis of a Clinical Trial. Journal of Medical Internet Research. https://doi.org/10.2196/53562

**51.** Zhu, X. T., Cheerman, H., Cheng, M., et al. (2025). Designing VR Simulation System for Clinical Communication Training with LLMs-Based Embodied Conversational Agents. Extended Abstracts of the CHI Conference on Human Factors in Computing Systems. https://doi.org/10.1145/3706599.3719693

**52.** Guhan, P., Awasthi, N., McDonald, K., et al. (2025). Developing a Machine Learning-Based Automated Patient Engagement Estimator for Telehealth: Algorithm Development and Validation Study. JMIR Formative Research. https://doi.org/10.2196/46390

**53.** Gibson, J., Can, D., Xiao, B., et al. (2016). A Deep Learning Approach to Modeling Empathy in Addiction Counseling. INTERSPEECH 2016. https://doi.org/10.21437/Interspeech.2016-554

**54.** Matthews, A., Lerman, A., Beach, M., et al. (2025). "It's like having that supervisor in the room": Examining AI as a reflective partner. Psychotherapy Research. https://doi.org/10.1080/10503307.2025.2569047

**55.** Sheperis, D., & Sadeh-Sharvit, S. (2023). Using AI-supported supervision in a university telemental health training clinic. Journal of Technology in Counselor Education and Supervision. https://doi.org/10.61888/2692-4129.1093

**56.** Vanin, A., Bolshev, V., Panfilova, A., et al. (2025). Psychotherapist remarks' ML classifier: insights from LLM and topic modeling application. Frontiers in Psychiatry. https://doi.org/10.3389/fpsyt.2025.1608163

**57.** Creed, T. A., Kuo, P. B., Oziel, R., et al. (2021). Knowledge and Attitudes Toward an Artificial Intelligence-Based Fidelity Measurement in Community Cognitive Behavioral Therapy Supervision. Administration and Policy in Mental Health. https://doi.org/10.1007/s10488-021-01167-x

**58.** Wang, T., Yuan, Y., & Zeng, T. (2025). Creating AI-Powered Intelligent Tutoring Systems for Teaching Psychotherapeutic Techniques in Mental Health Education. 2025 IEEE 5th International Conference on ICT in Business, Industry & Government (ICTBIG). https://doi.org/10.1109/ICTBIG68706.2025.11323910

**59.** Cruz, R., Felix Romero, V., Rosas, M., Tzek, D. P., & Meza, I. (2025). Automatic feedback through natural language processing using a chatbot-based simulated patient (PEPE) for the training of mental health professionals. Journal of Artificial Intelligence and Computing Applications, 3(2), 33–37. https://doi.org/10.5281/zenodo.17227862

**60.** Held, P., Pridgen, S. A., Szoke, D. R., et al. (2025). Clinician Perceptions of Socrates 2.0: A Multi-Agent Artificial Intelligence Tool to Facilitate Socratic Dialogue. Cognitive and Behavioral Practice. https://doi.org/10.1016/j.cbpra.2025.10.001

**61.** Gill, S., & Galletly, C. (2025). 'I, robot, can help you'. Applications of Generative Artificial Intelligence in RANZCP psychiatry training. Australasian Psychiatry. https://doi.org/10.1177/10398562251344470

**62.** Kuo, P., Mehta, M., Hashtpari, H., et al. (2024). Identification of cultural conversations in therapy using natural language processing models. Psychotherapy. https://doi.org/10.1037/pst0000542

**63.** Lin, B. (2022). SupervisorBot: NLP-annotated real-time recommendations of psychotherapy treatment strategies with deep reinforcement learning. arXiv. https://doi.org/10.48550/arXiv.2208.13077

**64.** Lin, B., Cecchi, G. A., Bouneffouf, D., et al. (2023). Psychotherapy AI Companion with Reinforcement Learning Recommendations and Interpretable Policy Dynamics. The Web Conference. https://doi.org/10.1145/3543873.3587623

**65.** Wu, Z. X., Helaoui, R., Recupero, D. R., et al. (2022). Towards Automated Counselling Decision-Making: Remarks on Therapist Action Forecasting on the AnnoMI Dataset. INTERSPEECH 2022. https://doi.org/10.21437/Interspeech.2022-506

**66.** Imel, Z. E., Pace, B., Pendergraft, B., et al. (2024). Machine Learning-Based Evaluation of Suicide Risk Assessment in Crisis Counseling Calls. Psychiatric Services. https://doi.org/10.1176/appi.ps.20230648

**67.** Lin, B., Bouneffouf, D., Landa, Y., Jespersen, R., Corcoran, C., & Cecchi, G. (2024). COMPASS: Computational Mapping of Patient-Therapist Alliance Strategies with Language Modeling. arXiv. https://arxiv.org/abs/2402.14701

**68.** Masri, R. Y., Jani, H. M., & Chong, A. T. Y. (2008). Expert system approach in diagnosing mental health: A proposal. 2008 International Symposium on Information Technology. https://doi.org/10.1109/ITSIM.2008.4631566

**69.** Longobardi, T., Sperandeo, R., Albano, F., et al. (2018). Co-regulation of the voice between patient and therapist in psychotherapy: machine learning for enhancing the synchronization of the experience of anger emotion. 2018 9th IEEE International Conference on Cognitive Infocommunications (CogInfoCom). https://doi.org/10.1109/CogInfoCom.2018.8639875

**70.** İçen, S., & Köken, A. H. (2025). Artificial intelligence guidance in ethically challenging clinical scenarios in child and adolescent psychiatry: A qualitative study in the context of Türkiye. BMC Medical Ethics, 26, Article 183. https://doi.org/10.1186/s12910-025-01323-0

**71.** Magill, M., Mastroleo, N. R., & Martino, S. (2022). Technology-Based Methods for Training Counseling Skills in Behavioral Health: A Scoping Review. Journal of Technology in Behavioral Science. https://doi.org/10.1007/s41347-022-00252-8

**72.** Abdou, M. M., Sahi, R. S., Hull, T. D., et al. (2025). Leveraging Large Language Models to Estimate Clinically Relevant Psychological Constructs in Psychotherapy Transcripts. Computational Psychiatry. https://doi.org/10.5334/cpsy.141

**73.** Kuo, P., Tanana, M. J., Goldberg, S. B., et al. (2024). Machine-learning-based prediction of client distress from session recordings. Clinical Psychological Science. https://doi.org/10.1177/21677026231172694

Classification framework cited in the table note: Bernard, J. M., & Goodyear, R. K. (2019). Fundamentals of Clinical Supervision (6th ed.). Pearson.
