# Supplementary material for: Artificial Intelligence in Clinical Supervision and Training of Psychologists and Other Mental Health Professionals: A Scoping Review
Source: Clin Psychol Psychother. 2026 Nov 9;33(6):e70359. doi: 10.1002/cpp.70359 (PMC13748793; doi:10.1002/cpp.70359)
Supplement: Supplementary file 2 [file CPP-33-e70359-s002.docx]

Ovid MEDLINE(R) ALL <1946 to March 21, 2025>

1	PSYCHOTHERAPY/ or PSYCHOTHERAPY, GROUP/	71595

2	(psychotherapy or (psychological adj (therap* or intervention))).tw.	47682

3	COGNITIVE THERAPY/	32764

4	((cogniti$ adj3 behavio$) or CBT).tw.	74363

5	(cognitive resructur* or cognitive processing).tw.	7253

6	CBASP.tw.	109

7	BEHAVIOR THERAPY/	31367

8	(behavio* adj3 (therap* or psychotherap* or intervention* or program* or training)).tw.	77237

9	behavio* activat*.tw.	2949

10	COUNSELING/	41243

11	counsel*.tw.	149556

12	MOTIVATIONAL INTERVIEWING/	2872

13	MOTIVATION/	84820

14	(motivational adj3 (interview* or intervention* or therap* or psychotherap* or program* or training)).tw.	7964

15	NONDIRECTIVE THERAPY/	453

16	(analytic and (cogniti* or therap* or psychotherapy*)).tw.	9838

17	(acceptance adj2 commitment).tw.	2202

18	compass* foc*.tw.	275

19	(dialectic* or DBT).tw.	7334

20	mind train*.tw.	111

21	(mindful* or meditation or relaxation).tw.	165994

22	rational emotive.tw.	317

23	(reality adj (therap* or psychotherap*)).tw.	262

24	or/1-23	652237

25	((Psychotherap* or Therapist* or "behavioral health practitioners" or psychologist*) adj3 (supervision* or trainee* or education or training or teaching or learning or information or course* or program* or Simulat* or "learn curve" or Mentoring or "role plays" or "role-playing" or "screening optimization")).ti,ab.	6528

26	Simulation Training/ or Mentoring/ or Teaching/ or Staff Development/ or Inservice Training/ or Education/	113561

27	Psychology/	24467

28	Psychology.sh.	24467

29	or/27-28	24467

30	29 and 26	954

31	(supervision* or trainee* or education or training or teaching or learning or information or course* or program* or Simulat* or "learn curve" or Mentoring or "role plays" or "role-playing" or "screening optimization").ti,ab.	5296600

32	31 or 26	5329708

33	31 or 32	5329708

34	((("artificial intelligence" or AI) adj2 (chat* or generat*)) or GenAI or ((large or natural or generative or machine or deep learning) adj3 (language or text) adj3 model*) or AlexaTM or (Amazon* and Alexa) or Anthropic or Bard or Bardeen or BERT or "Bing chat" or BioGPT or BLOOM or BloombergGPT or Cerebras-GPT or ChatGPT* or "Chat GPT" or chatbot* or Chatsonic or Chinchilla or Claude or DALL-E or EinsteinGPT or Ernie or Falcon or Galactica or "Generative Fill" or "GitHub Copilot" or GLaM or "Google* Assistant" or "Google* Bard" or "Google* Gemini" or Gopher or GPT-1 or GPT-2 or GPT-3* or GPT-4* or GPTNeo or GPT-NEoX or GPT-J* or "IBM Watson" or LaMDA or LLaMA or "Megatron-Turing NLG" or "Microsoft* Bing" or "Microsoft* Copilot" or Midjourney or Minerva or NeevaAI or Nvidia or OpenAI or "Open AI" or OpenAssistant or PaLM or PanGu-E or PathAI or "Path AI" or Perplexity or "pre-trained transformer*" or "pretrained transformer*" or (Apple* and Siri) or SlackGPT or "Stable Diffusion" or StyleGAN or Synthesia or XLNet or YaLM 100B or YouChat).mp.	61638

35	artificial intelligence/	53366

36	30 or 33	5329708

37	34 or 35	111453

38	36 and 37	44447

39	30 and 37	1

40	24 and 32	218071

41	40 or 25	220711

42	41 and 37	738

43	39 or 42	738
